# Supplementary material for: The Cytosolic Acetoacetyl-CoA Thiolase TaAACT1 Is Required for Defense against Fusarium pseudograminearum in Wheat
Source: Int J Mol Sci. 2023 Mar 24;24(7):6165. doi: 10.3390/ijms24076165 (PMC10094598; doi:10.3390/ijms24076165)
Supplement: Supplementary file 1 [file ijms-24-06165-s001.zip › ijms-2290675-supplementary.pptx]

## Slide 1
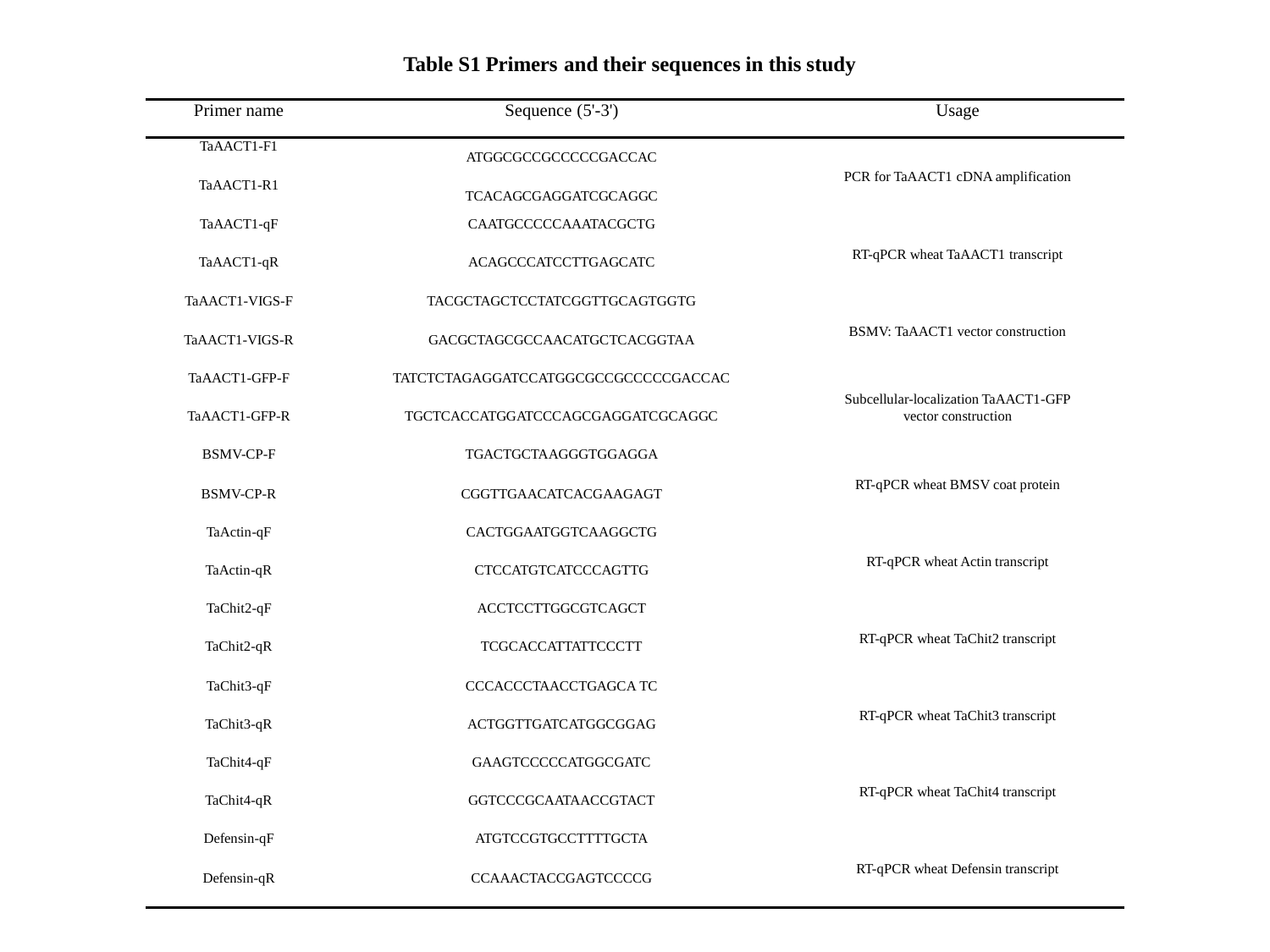

## Slide 2
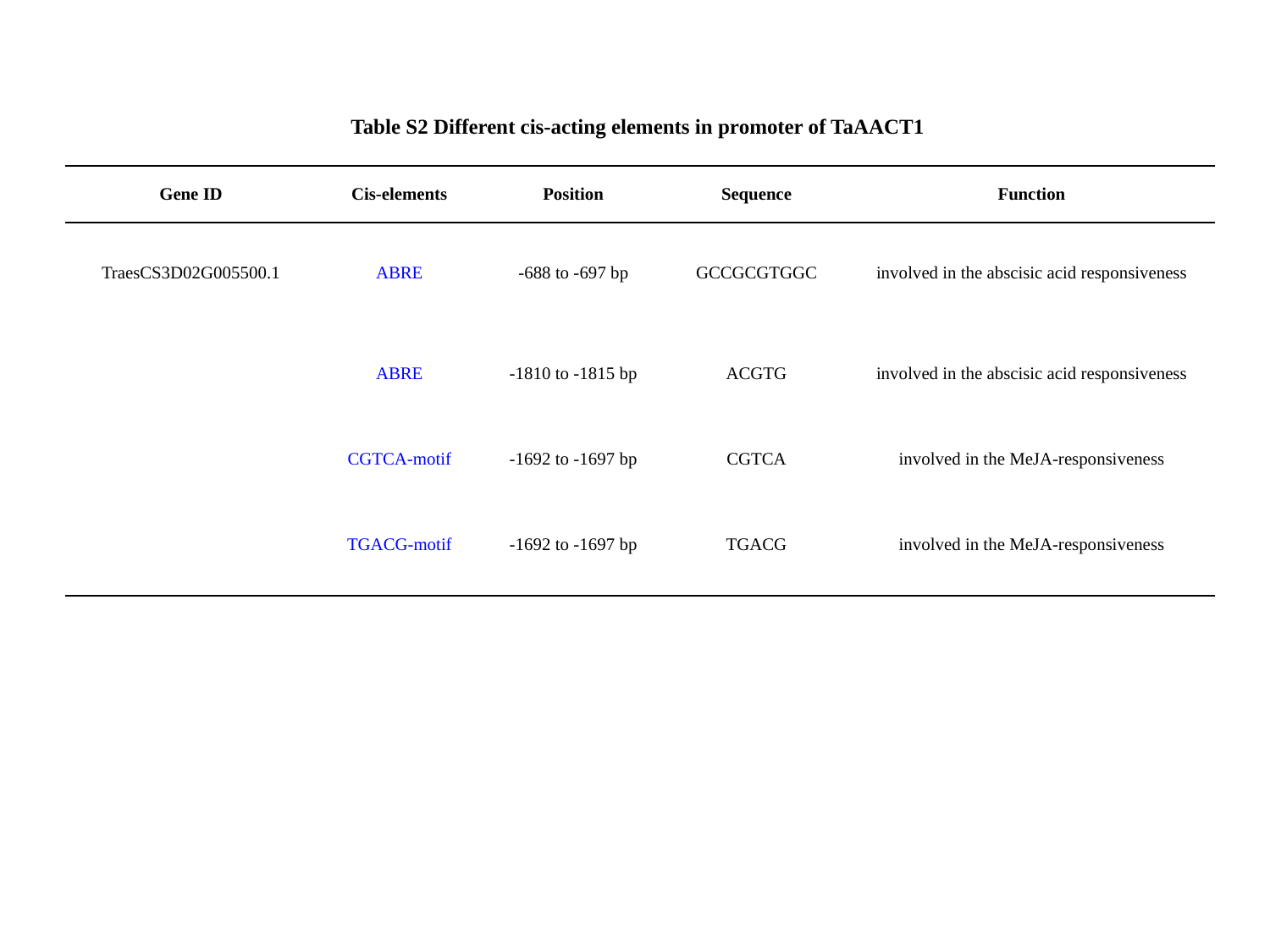

Table S2 Different cis-acting elements in promoter of TaAACT1
| Gene ID | Cis-elements | Position | Sequence | Function |
| --- | --- | --- | --- | --- |
| TraesCS3D02G005500.1 | ABRE | -688 to -697 bp | GCCGCGTGGC | involved in the abscisic acid responsiveness |
| | ABRE | -1810 to -1815 bp | ACGTG | involved in the abscisic acid responsiveness |
| | CGTCA-motif | -1692 to -1697 bp | CGTCA | involved in the MeJA-responsiveness |
| | TGACG-motif | -1692 to -1697 bp | TGACG | involved in the MeJA-responsiveness |
